# Supplementary material for: Evaluation of health-related quality of life using EQ-5D in China during the COVID-19 pandemic
Source: PLoS One. 2020 Jun 18;15(6):e0234850. doi: 10.1371/journal.pone.0234850 (PMC7302485; doi:10.1371/journal.pone.0234850)
Supplement: S1 File — (DOC) [file pone.0234850.s001.doc]

长治市居民“新冠”期间生活质量调查问卷

尊敬的居民：

您好！

自从2019年12月在我国湖北武汉市爆发新型冠状病毒肺炎以来，全国各地均采取了严格的管理措施以控制疾病的扩散。我们调查的目的在于了解您在这一特殊时期内生活质量的情况，希望能够得到您的配合，如果您同意填写此问卷，我们就视为您了解了我们的调查内容。此问卷为匿名问卷，所有信息将仅用于此研究，并进行保密处理。填写问卷大约需要5分钟时间。

1. 您的性别：£男 £女
2. 您的年龄： （岁）
3. 您的婚姻状况：£已婚 £未婚 £离婚 £丧偶 £分居
4. 您的职业： £在职 £退休 £居家 £失业 £无业
5. 您的文化程度：£小学及以下 £初中 £高中 £大学及以上
6. 您的家庭收入在本地处于什么水平：£最高 £较高 £一般 £偏低 £最低
7. 您是否担心自己会感染该病： £非常担心 £担心 £不担心 £完全不担心
8. 在过去6个月内您是否患有慢性疾病：£是 £否。

如果您患有慢性疾病，是以下类型中的哪些（可以多选）（ ）

£高血压 £糖尿病 £血脂异常 £心脏病（冠心病、心律失常、心脏瓣膜病） £脑血管疾病 £周围血管疾病 £慢性阻塞性肺病 £支气管扩张 £哮喘 £肺纤维化 £病毒性肝炎 £脂肪肝 £其他慢性肝病 £胆囊疾病 £胃炎 £胃溃疡 £十二指肠溃疡 £慢性肠炎 £肠息肉 £慢性疼痛 £痛风 £关节炎 £甲状腺疾病 £肾脏疾病 £膀胱疾病 £子宫卵巢疾病

£前列腺疾病 £中耳炎 £失聪 £青光眼 £白内障 £其他眼部疾病 £皮肤疾病 £血液疾病 £帕金森症 £其他疾病

1. 疫情对您在以下方面有无影响程度

|  | 极大负面影响 | 有负面影响 | 一点负面影响 | 无影响 | 一点正面影响 | 有正面影响 | 极大正面影响 |
| --- | --- | --- | --- | --- | --- | --- | --- |
| 社交活动 |  |  |  |  |  |  |  |
| 日常生活和行程安排 |  |  |  |  |  |  |  |
| 睡眠 |  |  |  |  |  |  |  |
| 饮食 |  |  |  |  |  |  |  |
| 运动锻炼 |  |  |  |  |  |  |  |
| 工作稳定性 |  |  |  |  |  |  |  |
| 个人学习、创作 |  |  |  |  |  |  |  |
| 经济收入 |  |  |  |  |  |  |  |
| 与父母关系 |  |  |  |  |  |  |  |
| 与朋友的关系 |  |  |  |  |  |  |  |
| 夫妻关系 |  |  |  |  |  |  |  |
| 子女教育 |  |  |  |  |  |  |  |

欧洲生存质量测定量表（EQ-5D）

请在下列各组选项中，指出哪一项叙述最能反映您今天的健康状况，并在空格内打√。


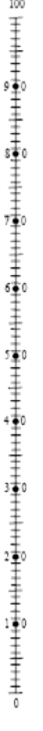


心目中最好的健康状况

心目中最差的健康状况

行动

我可以四处走动，没有任何困难 □

我行动有些不方便 □

我卧病在床 □

自己照顾自己

我能自己照顾自己，没有任何困难 □

我在洗脸、刷牙、洗澡或穿衣方面有些困难 □

我无法自己洗脸，刷牙，洗澡或穿衣 □

日常活动（如工作，学习，家务事，家庭或休闲活动）

我能进行日常活动，没有任何困难 □

我在进行日常活动方面有些困难 □

我无法进行日常活动 □

疼痛/不舒服

我没有任何疼痛或不舒服 □

我觉得中度疼痛或不舒服 □

我觉得极度疼痛或不舒服 □

焦虑/沮丧

我不觉得焦虑或抑郁 □

我觉得中度焦虑或抑郁 □

我觉得极度焦虑或抑郁 □

为了帮助您反映健康状况的好坏，我们画了一个刻度尺（有点像温度计），在这刻度尺上，100代表您心目中最好的状况，0代表您心目中最差的状况。

请在右边的刻度尺上标出您今天的健康状况。请从下面方格中划出一条线，连到刻度尺上最能代表您今天健康状况好坏的那一点。

非常感谢您的参与！
