# Supplementary material for: Evaluation of health-related quality of life using EQ-5D in China during the COVID-19 pandemic
Source: PLoS One. 2020 Jun 18;15(6):e0234850. doi: 10.1371/journal.pone.0234850 (PMC7302485; doi:10.1371/journal.pone.0234850)
Supplement: S2 File — (DOCX) [file pone.0234850.s002.docx]

**Questionnaire for the Quality of Life of Residents of the Changzhi During the "COVID-19"**

Ladies and Gentalment:

Since the outbreak of “COVID-19” in December 2019 in Wuhan, Hubei province, China , the strict management measures were adopted to control the spread of this disease in our country. The aim of the survey is to know about your quality of life during this particular period, and we hope to get your cooperation. If you agree to fill out this questionnaire, we think you understand the content of our survey. This questionnaire is anonymous, all information will only be used for study and be treated confidentially. It takes about 5 minutes to complete it. Thank you very much.

1. Your gender: □male □female

2. Your age: _____(year)

3. Your marital status: □married □unmarried □divorced □widowed □separated

4. Your occupation: □In service □retirement □in home □lose job □no job

5. Your education level: □primary school education and below □junior middle school

□high school □university and above

1. What is the level of your household income locally?

□highest □high □normal □low □lowest

1. Are you worried that you will contract this disease?

□very worried □worried □not worried □not worried at all

8. Have you suffered from a chronic disease in the past 6 months: □Yes □No

If you have a chronic disease, which of the following types (multiple choices) :

□hypertension □diabetes □dyslipidemia □cerebrovascular disease

□heart disease (coronary heart disease, arrhythmia, heart valve disease) □bronchiectasis

□peripheral vascular disease □chronic obstructive pulmonary □asthma

□pulmonary fibrosis □viral hepatitis □fatty liver □other chronic liver diseases

□gallbladder disease □gastritis □gastric ulcer □duodenal ulcer □chronic enteritis □intestinal polyps □chronic pain □gout □arthritis □thyroid disease □kidney disease □bladder disease □uterine and ovarian disease □prostate disease □otitis media

□deafness□glaucoma □cataract □other eye diseases □skin diseases

□blood diseases □parkinson's disease □other diseases

9. The influence of the “COVID-19” on you in the following aspects:

| contents | great negative | negative | a little negative | no | a little positive | positive | great  positive |
| --- | --- | --- | --- | --- | --- | --- | --- |
| social activities |  |  |  |  |  |  |  |
| daily life and schedule |  |  |  |  |  |  |  |
| sleep |  |  |  |  |  |  |  |
| diet |  |  |  |  |  |  |  |
| exercise |  |  |  |  |  |  |  |
| working stability |  |  |  |  |  |  |  |
| personal learning or creation |  |  |  |  |  |  |  |
| income |  |  |  |  |  |  |  |
| Relationship with parents |  |  |  |  |  |  |  |
| relationship with friends |  |  |  |  |  |  |  |
| marriage relationship |  |  |  |  |  |  |  |
| children education |  |  |  |  |  |  |  |

**European five-dimensional health scale(EQ-5D)**

Please indicate in the following set of options which statement best reflects your health status today, and type√ in the space.


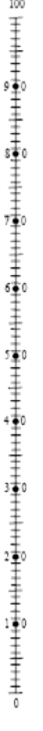


The best health in mind

The worst health in mind

**Mobility**

I can mobile around without any difficulty. □

I am a little inconvenient. □

I'm sick in bed. □

**Self-Care**

I can take care of myself without any difficulty. □

I have some difficulties in washing my face, brushing my teeth, bathing or dressing. □

I can’t wash my face, brush my teeth, take a bath or dress myself. □

**Usual activities (such as work, study, housework, family or leisure activities)**

I can do usual activities without any difficulty. □

I have some difficulties in usual activities. □

I can't do usual activities. □

**Pain/Discomfort**

I don't have any pain or discomfort. □

I feel moderate pain or discomfort. □

I feel extreme pain or discomfort. □

**Anxiety/Depression**

I don't feel anxious or depressed. □

I feel moderate anxiety or depression. □

I feel extremely anxious or depressed. □

为了帮助您反映健康状况的好坏，我们画了一个刻度尺（有点像温度计），在这刻度尺上，100代表您心目中最好的状况，0代表您心目中最差的状况。

请在右边的刻度尺上标出您今天的健康状况。请从下面方格中划出一条线，连到刻度尺上最能代表您今天健康状况好坏的那一点。

In order to help you reflect your health, we drew a scale (a little like a thermometer). On this scale, 100 represents the best health situation in your mind, and 0 represents the worst health situation in your mind.

Please mark your health situation on the scale on the right. Please draw a line from the box below and connect it to the scale that best represents your health situation today.

**Thank you very much for your participation!**
