# Supplementary material for: Evaluation of health-related quality of life using EQ-5D in China during the COVID-19 pandemic
Source: PLoS One. 2020 Jun 18;15(6):e0234850. doi: 10.1371/journal.pone.0234850 (PMC7302485; doi:10.1371/journal.pone.0234850)
Supplement: Attachment — Submitted filename: Response to Reviewers.docx [file pone.0234850.s004.docx]

**Our responds to the reviewer’s comments：**

**Reply to reviewer**

We thank you very much for the constructive comments and suggestions on our manuscript. We have corrected our manuscript according to the comments.

1. In Introduction, the sentence “The EuroQol (EQ-5D) is perhaps the most commonly used by researchers” is too strong. Please tone down the statement. If the authors want to keep this statement, please provide evidence. For example, how many papers have been using EQ-5D and how many papers have been using other quality of life instruments?

Reply: we agree and accept your suggestion, “The EuroQol (EQ-5D) is perhaps the most commonly used by researchers.” has been deleted, and description showed in the manuscript as follows "The EQ-5D is a simple but widely used instrument based on characteristic that can be used measured generic population based on utility".

2. As the authors want to study in quality of life, they should firstly introduce different types of quality of life measures. From the aspect of “condition”, quality of life instruments can be classified into “condition-specific instrument” and “generic instrument”. From the aspect of “psychometrics”, quality of life instruments can be classified into “psychometric instrument” and “utility instrument”. The authors should clearly introduce these different types and clearly let the readers know that the EQ-5D is a generic instrument based on utility. Please refer to the following.

***References on “condition”**

Lin, C.-Y., Lee, T.-Y., Sun, Z.-J., Yang, Y.-C., Wu, J.-S., & Ou, H.-t. (2017). Development of diabetes-specific quality of life module to be in conjunction with the World Health Organization Quality of Life Scale Brief Version (WHOQOL-BREF). Health & Quality of Life Outcomes, 15, 167.

Lin, C.-Y., Hwang, J.-S., Wang, W.-C., Lai, W.-W., Su, W.-C., Wu, T.-Y., Yao, G., & Wang, J.-D. (2019). Psychometric evaluation of the WHOQOL-BREF, Taiwan version, across five kinds of Taiwanese cancer survivors: Rasch analysis and confirmatory factor analysis. Journal of the Formosan Medical Association, 118(1), 215-222.

Lin, C.-Y. (2018). Comparing quality of life instruments: Sizing Them Up versus PedsQL and Kid-KINDL. Social Health & Behavior, 1, 42-47.

Pakpour, A. H., Chen, C.-Y., Lin, C.-Y., Strong, C., Tsai, M.-C., & Lin, Y.-C. (2019). The relationship between children's overweight and quality of life: A comparison of Sizing Me Up, PedsQL, and Kid-KINDL. International Journal of Clinical and Health Psychology, 19(1), 49-56.

***Reference on “psychometrics”**

Lin, H. W., Li, C. I., Lin, F. J., Chang, J. Y., Gau, C. S., Luo, N., Pickard, A. S., Ramos Goñi, J. M., Tang, C. H., & Hsu, C. N. (2018). Valuation of the EQ-5D-5L in Taiwan. PloS One, 13(12), e0209344.

Lee, H. Y., Hung, M. C., Hu, F. C., Chang, Y. Y., Hsieh, C. L., & Wang, J. D. (2013). Estimating quality weights for EQ-5D (EuroQol-5 dimensions) health states with the time trade-off method in Taiwan. Journal of the Formosan Medical Association, 112(11), 699-706.

Reply: we thank very much for your suggest on introduction section. We read carefully literatures you mentioned , and add related contents in the manuscript as follows: Some scales have been widely developed to measure HRQOL. Generic instrument of HRQOL, for example: the World Health Organization Quality of Life (WHOQOL-100) , can be used to compare HRQOL across various diseases/conditions and can be considered useful for different populations to assess the impact of various interventions on QOL. Condition-specific instrument of HRQOL (e.g.EORTCQLQ2-C30) can only be used to compare HRQOL specific diseases/conditions for specific populations (e.g. cancer or diabetes). On the other hand, the quality of life should be measured on a utility scale on which 1.0 corresponds to full health and 0.0 corresponds to death. Therefore, HRQOL scales can be classified into psychometric instrument (e.g. SF-36 ), and utility instrument (e.g. the Health Utilities Index). The EQ-5D is a simple but widely used instrument based on characteristic that can be used measured generic population based on utility.

3. Regarding the 1500 surveys, how did the authors perform the random selection? Please provide the process. For example, did the authors obtain a list before sending out the surveys? If yes, how and where did the authors obtain the list? How was the representativeness of the list? Did the list include all the residents in Changzhi city?

Reply: because the survey conducted in the period of epidemic, an online-based survey was developed and participants were recruited via social media (e.g., We-Chat). A statement about informed consent was included with the questionnaire, and returning the questionnaire was considered to constitute provision of informed consent. At March 10, 1500 questionnaire were returned by respondents. Of these, 215 respondents were not residents that lived in Changzhi city according to the location.146 were deemed unusable due to using time is less than 100 seconds. The left 1139 were deemed usable.

4. In Results, the subheading of Regression analysis is misleading. Specifically, regression analysis gives an impression of “linear regression”. Therefore, the authors should explicitly mention “logistic regression” for the subheading.

Reply: we agree and accept your suggestion, The title of Table 3 has been corrected “Multivariate logistic regression analysis results on the relationships between 5 dimensions of EQ-5D and influence factors”.

5. Table 3. The heading of “CI 95 (Exp B)” is confusing. Please remove (Exp B). Also, it is unclear why there is a * sign before 1.798 for the marital variable. The title of Table 3 should clearly state whether this is multivariate logistic regression model or univariate logistic regression model.

Reply: we agree and accept your suggestion, the heading of “CI 95 (Exp B)” has been corrected “ 95%CI”.“ * sign before 1.798”is a mistake, we has been corrected it to"-1.798" in our manuscript. The title of Table 3 has been corrected “Multivariate logistic regression analysis results on the relationships between 5 dimensions of EQ-5D and influence factors”.

6. Following the prior comment, it is unclear whether the authors conducted multivariate logistic regression or univariate logistic regression. This needs to be clear.

Reply: we think our description about multivariate logistic regression is unclear, so ,we corrected the section as follows in our manuscript: Each dimension of EQ-5D have been dichotomized, and have been used dependent variable. Sex, age, marital status, employment status, chronic disease condition, education level, family income level in the local, worry about get COVID-19, epidemic effects have been included as independent variables, multivariate logistic regression models were conducted, only those variables which exerted a significant relationship with any dimension from EQ-5D were reported in Table 3.

7. As the authors conducted a series of related analyses, they should adjust the p-values.

Reply: we agree and accept your suggestion, so we made the following modifications: in Table2, the row of "some" and "extreme" were merged into one row, *x*^2^ test and Fisher's exact test were conducted again, *p* value was obtained again, the result have been showed in Table2.

8. The authors should mention a limitation that, apart from the EQ-5D, all other measures used in the present study did not have been tested for psychometric properties. In particular, the measure on worry of COVID-19 seems to be developed by the authors themselves. Thus, the authors should acknowledge in the present study that there are available psychological distress instruments on COVID-19. Indeed, the authors found that anxiety/depression is the second frequently reported problem. Encouraging future studies using appropriate instruments on COVID-19 (e.g., the Fear of COVID-19 Scale and the COVID Stress Scales) is needed. Please see and cite the following references for discussion.

Ahorsu, D. K., Lin, C. Y., Imani, V., Saffari, M., Griffiths, M. D., & Pakpour, A. H. (2020). The Fear of COVID-19 Scale: Development and initial validation. International Journal of Mental Health and Addiction. Advance online publication. doi: 10.1007/s11469-020-00270-8.

Sakib, N., Mamun, M. A., Bhuiyan, A. K. M. I., Hossain, S., Mamun, F. A., Hosen, I., … Pakpour, A. H. (2020). Psychometric validation of the Bangla Fear of COVID-19 Scale: Confirmatory factor analysis and Rasch analysis. International Journal of Mental Health and Addiction. Advance online publication. doi: 10.1007/s11469-020-00289-x.

Satici, B., Gocet-Tekin, E., Deniz, M. E., & Satici, S. A. (2020). Adaptation of the Fear of COVID-19 Scale: Its association with psychological distress and life satisfaction in Turkey. International Journal of Mental Health Addiction. Advance online publication. doi: 10.1007/s11469-020-00294-0.

Soraci, P., Ferrari, A., Abbiati, F.A., Del Fante, E., De Pace, R., Urso A. Griffiths, M.D. (2020). Validation and psychometric evaluation of the Italian version of the Fear of COVID-19 Scale. International Journal of Mental Health and Addiction. Advance online publication. doi: 10.1007/s11469-020-00277-1.

Taylor, S., Landry, C., Paluszek, M., Fergus, T. A., Mckay, D., Asmundson, G. J. G. (2020). Development and initial validation of the COVID Stress Scales. Journal of Anxiety Disorders. Advance online publication. doi: 10.1016/j.janxdis.2020.102232.

Reply: we thank very much for your suggest on limitation section. We read literatures you mentioned carefully, and add related contents in the limitation section as follows: Lastly, the measure on worry of COVID-19 developed by ourselves in our study, but psychological distress instruments on COVID-19 has been developed by Ahorsu and Taylor in the recent study. So, it is necessary to use appropriate instruments on COVID-19 (e.g., the Fear of COVID-19 Scale and the COVID Stress Scales) to measure mental health in the future study.

9. Lastly, I think that the authors cannot claim to study the “impact of COVID-19 epidemic on quality of life” throughout the manuscript. The present study is a cross-sectional study and cannot provide such causality statement. Please revise all such statements.

Reply: we thank you very much for your constructive comments, and corrected the conclusion as follows " The risk of pain/discomfort and anxiety/depression in general population in China raised significantly with aging, with chronic disease, lower income, epidemic effects, worried about get COVID-19 during the COVID-19 pandemic." All statements in our manuscript has been deleted and corrected.

Once again, thank you very much for your constructive comments and suggestions which would help us both in English and in depth to improve the quality of the paper.
